# Carotid Body Type-I Cells Under Chronic Sustained Hypoxia: Focus on Metabolism and Membrane Excitability

**DOI:** 10.3389/fphys.2018.01282

**Published:** 2018-09-19

**Authors:** Raúl Pulgar-Sepúlveda, Rodrigo Varas, Rodrigo Iturriaga, Rodrigo Del Rio, Fernando C. Ortiz

**Affiliations:** ^1^Instituto de Ciencias Biomédicas, Facultad de Ciencias de la Salud, Universidad Autónoma de Chile, Santiago, Chile; ^2^Facultad de Ciencias de la Salud, Universidad Autónoma de Chile, Talca, Chile; ^3^Laboratorio de Neurobiología, Facultad de Ciencias Biológicas, Pontificia Universidad Católica de Chile, Santiago, Chile; ^4^Laboratory of Cardiorespiratory Control, Department of Physiology, Pontificia Universidad Católica de Chile, Santiago, Chile; ^5^Centro de Envejecimiento y Regeneración, Pontificia Universidad Católica de Chile, Santiago, Chile; ^6^Centro de Excelencia en Biomedicina de Magallanes, Universidad de Magallanes, Punta Arenas, Chile

**Keywords:** carotid body, chronic hypoxia, membrane depolarization, ion channels, TASK-like channel

## Abstract

Chronic sustained hypoxia (CSH) evokes ventilatory acclimatization characterized by a progressive hyperventilation due to a potentiation of the carotid body (CB) chemosensory response to hypoxia. The transduction of the hypoxic stimulus in the CB begins with the inhibition of K+ currents in the chemosensory (type-I) cells, which in turn leads to membrane depolarization, Ca^2+^ entry and the subsequent release of one- or more-excitatory neurotransmitters. Several studies have shown that CSH modifies both the level of transmitters and chemoreceptor cell metabolism within the CB. Most of these studies have been focused on the role played by such putative transmitters and modulators of CB chemoreception, but less is known about the effect of CSH on metabolism and membrane excitability of type-I cells. In this mini-review, we will examine the effects of CSH on the ion channels activity and excitability of type-I cell, with a particular focus on the effects of CSH on the TASK-like background K+ channel. We propose that changes on TASK-like channel activity induced by CSH may contribute to explain the potentiation of CB chemosensory activity.

## Introduction

The carotid body (CB) is the main peripheral chemoreceptor in mammals. Natural stimuli such as hypoxemia, hypercapnia, and/or acidosis increase the firing rate of the petrosal ganglion (PG) sensory afferent neurons projecting to the cardiovascular and respiratory regions in the brain stem (Gonzalez et al., 1994).

The current model for hypoxic chemoreception states that hypoxia evokes a depolarization of CB type-I (glomus) cells, leading to an increment of intracellular Ca^2+^ and the subsequent release of one- or more-excitatory neurotransmitters to the nerve terminals of the PG neurons (Gonzalez et al., 1994; Iturriaga and Alcayaga, 2004; Nurse, 2005). It is well established that a key event in triggering the hypoxic response is the depolarization of the type-I cells (Buckler and Vaughan-Jones, 1994). In type-I cells from several species it has been found that hypoxia produces a fast and reversible inhibition of K^+^ currents (López-Barneo et al., 1988; López-López and González, 1992; Wyatt and Peers, 1995; Buckler, 1997) leading to a depolarization of type-I cells membrane and the consequence Ca^2+^entry, mainly throughout L-type Ca^2+^ channels (Fieber and McCleskey, 1993; Wyatt et al., 1994). Among several molecules present in type-I cells, acetylcholine (ACh) and adenosine triphosphate (ATP) meets most of the criteria to be consider excitatory transmitters in the pathway (Varas et al., 2003; Iturriaga and Alcayaga, 2004), while dopamine (DA), nitric oxide (NO), and endothelin-1 (ET-1) modulate the chemosensory process (Iturriaga and Alcayaga, 2004).

Mammals exposed to sustained hypoxia (i.e., high altitude) develop ventilatory acclimatization featured by a progressively hyperventilation, due to an augmented CB responsiveness to hypoxia (Bisgard, 2000). In addition to the enhanced CB chemosensory responses, chronic sustained hypoxia (CSH) for weeks or months induces angiogenesis in the CB along with type-I cell hypertrophy and hyperplasia (Heath et al., 1985). Most of the studies on CB hypoxia acclimatization have been focused on putative changes in transmitters or modulators of CB chemoreception. Interestingly, DA, NO, and ET-1 are up-regulated in the CB during the first week of chronic hypoxia (Bisgard, 2000). In addition, some reports indicate that CSH increases type-I cell excitability due to changes in K^+^ and Na^+^ channels expression. In spite of the efforts, the mechanisms by which CSH enhances CB chemosensory responses to hypoxia remains to be established. In this review, we will examine the effects of sustained hypoxia on CB metabolism and ion channels function, focusing on the type-I cell excitability.

## Electrical Properties of Type-I Cells

Type-I cells are small round-shaped cells (diameter ∼10 μm in the rat), with high input resistance of 5–6 GΩ (Duchen et al., 1988; Xu et al., 2005), and resting membrane potential ranging from −50 to −70 mV (Gonzalez et al., 1994). Regarding to the inward currents expressed by type-I cells, in the adult rabbit it has been reported a tetrodotoxin-sensitive, voltage-gated Na^+^ current with fast activation and inactivation kinetics (Duchen et al., 1988). However, in both adult and neonatal rat type-I cells, there are contradictory results regarding the expression of functional voltage gated Na^+^ channels (Fieber and McCleskey, 1993; Hempleman, 1995; Stea et al., 1995; Cáceres et al., 2007). Voltage-gated Ca^2+^ channels have been found in both rat and rabbit type-I cells (Fieber and McCleskey, 1993; Buckler and Vaughan-Jones, 1994; Wyatt et al., 1994). Most of the studies agree that L-type Ca^2+^ channel is the most abundant subtype of Ca^2+^ channel in type-I cells, however, in rabbit CB type-I cells, pharmacological evidences suggest the additional presence of N-, P/Q-type Ca^2+^ channels and a ω-conotoxin-resistent Ca^2+^ current (Overholt and Prabhakar, 1997, 1999). In addition, rat type-I cells express anionic currents (mainly Cl^−^) which are involved in the chemosensory transduction of acidic and hypercapnic stimuli (Carpenter and Peers, 1997; Iturriaga et al., 1998).

Type-I cells express a wide variety of K^+^ channels. Rabbit type-I cells express at least two voltage-gated, TEA-sensitive Ca^2+^ – independent K^+^ currents. One of these conductances correspond to an oxygen-sensitive voltage-gated K^+^ channel with a unitary conductance of ∼40 pS and an activation threshold around −40 to −30 mV (hereafter KO_2_; López-Barneo et al., 1988). The opening of this K^+^ channel is reversibly inhibited by hypoxia with an IP_50_ (PO_2_ at which 50% of maximal inhibition is reached) near to 5–10 mmHg. This inhibition by hypoxia depends on membrane potential: maximal inhibition (40% of control activity) is reached at 0 mV (López-Barneo et al., 1988). The KO_2_ channel has at least four closed states (C0–C4), one open state (O) and two inactivated states (I1–I2). Hypoxia induces both, stabilization of the “C0” state and promoting the channel from “O” state to inactivation state “I1” (Ganfornina and Lopez-Barneo, 1992).

Neonatal rat type-I cells express a maxi-K (BK) channel, whose opening depends on the PO_2_. This maxi-K channel has a large unitary conductance of ∼200 pS, it is blocked by charybdotoxin and activated by both membrane depolarization (threshold ranging from −40 to −20 mV) and by a rise in intracellular [Ca^2+^] above 100 nM (Peers, 1990; Wyatt and Peers, 1995). Wyatt and Peers (1995) found that hypoxia (PO_2_ = 5–10 mmHg) causes a reversible inhibition of this maxi-K current in cell-attached patch-clamp recordings. Later, Williams et al. (2004) proposed that the oxygen-sensitivity of the rat type-I cells is mediated by a heme oxygenase-2 (HO-2) associated with the maxi-K channel complex. However, this proposition was challenged by Ortega-Sáenz et al. (2006), when they found that hypoxic response of type-I cells remains intact in HO-2 null mice.

Interestingly, Buckler (1997) found that voltage gated K^+^ channel blockers (10 mM TEA plus 5 mM 4-aminopyridine and 20 nM charybdotoxin) failed to depolarize type-I cells and to modify intracellular [Ca^2+^]. However, in the presence of these K^+^ channel blockers, a hypoxic stimulus was able to evoke cell depolarization and a rise in intracellular Ca^2+^ levels. This background K^+^ current was inhibited by hypoxia with an IP_50_ ∼10 mmHg, reaching a maximal ∼70% inhibition during anoxia. Hypoxia stabilized the background K^+^ channel in its close state, but did not affect the opening/closing kinetics, or the open state duration (Williams and Buckler, 2004). This background K^+^ channel correspond to a TWIK-related acid-sensitive K^+^ channel (TASK), member of the two-pore domain K^+^ channel superfamily (Buckler et al., 2000). Additional evidences suggest the presence of TASK-1, TASK-2, TASK-3, TRAAK, and TREK (Buckler, 1997; Buckler et al., 2000; Yamamoto et al., 2002; Yamamoto and Taniguchi, 2006).

**FIGURE 1:**
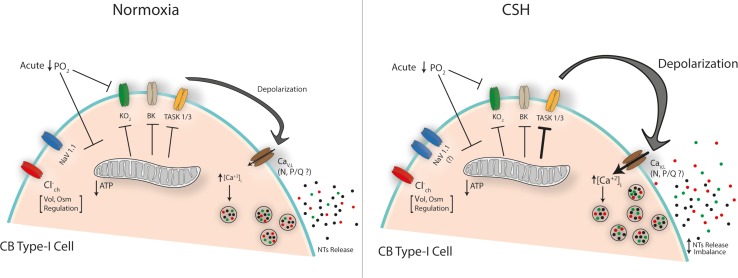
Proposed mechanisms of CB type-I cell hyperexcitability under chronic sustained hypoxia (CSH). The scheme depicts the main ion channels described in CB type-I cells. Acute fall in the PO_2_ inhibits K^+^ conductance(s) either directly on membrane components (i.e., KO_2_) or indirectly by reducing mitochondrial ATP production (‘metabolic hypothesis’) triggering a membrane depolarization that leads to the calcium-dependent neurotransmitters (NTs) release, initiating the chemosensory response to hypoxia. Under CSH an increased Na-current has been suggested [Nav1.1 subunit increased expression (Cáceres et al., 2007)] while a potentiated inhibition of a TASK-like K^+^-channel activity which induced a larger depolarization has been described (Ortiz et al., 2007, 2013). A major voltage-dependent calcium entry (mainly through L-type Ca-channels) is expected, which could explain the CB neurotransmitter release imbalance under CSH. Chloride channel(s) have been related with volume (Vol) and osmolarity (Osm) regulation but they would not participate in CSH mechanisms.

The discovery of oxygen-dependent voltage-gated K^+^ channels promptly leads to the suggestion that hypoxia inhibits K^+^ channels, which in turn produces type-I cells depolarization. Nevertheless, the resting membrane potential of type-I cells is quite stable at least 10 mV below the threshold activation of both maxi-K and KO_2_ channels. Therefore, it was hard to conciliate a possible role of these K^+^ channels in initiating the depolarization in response to hypoxia in type-I cells. Since background K^+^ channels (Buckler, 1997; Buckler et al., 2000) are active at resting conditions, the closure of these channels may explain the initiation of the depolarization evoked by hypoxia. The precise mechanism by which acute hypoxia is sensed remains controversial, but is clear that TASK, maxi-K, and KO_2_ channels plays a key role in the depolarization required for the neurotransmitters release from type-I cells in response to hypoxia (**Figure 1**).

## Chronic Sustained Hypoxia (Csh)

Hypoxic-hypoxia (a PO_2_ fall), could be classified as acute (seconds to minutes) or chronic (days to years). Acute hypoxia produced CB chemosensory excitation that evokes a reflex hyperventilation. CB increases its size in response to chronic sustained hypoxia (CSH) – to differentiate it from the *intermittent* paradigm – due to both increased number of cells and enhanced cell bodies diameter (Pequignot et al., 1984; Mills and Nurse, 1993). In this line, Pardal et al. (2007) have reported that in mice exposed to 10% O_2_ for 7 days, a subpopulation of type-II cells acts like stem-cells, differentiating into new type-I cells. This mechanism may explain why CB hyperplasia is induced by CSH (Pardal et al., 2007). Likewise, several evidences suggest an important role of type-II cells in CB adaptation to different paradigms of chronic hypoxia (see Leonard et al., 2018 for a recent revision on this subject). The progressively increases in ventilation elicited by CSH, it is known as ventilatory acclimatization (Bisgard, 2000). Ventilatory acclimatization induced by CSH depends on the potentiation of the CB chemosensory response to hypoxia in several species (Bouverot et al., 1973; Olson and Dempsey, 1978; Smith et al., 1986; Vizek et al., 1987; Di Giulio et al., 2003). In this section we summarized some evidences concerning possible mechanisms behind this phenomenon.

### Increased Levels of NO and ET-1 in the CB

Chronic sustained hypoxia increases the levels of NO in the CB (Ye et al., 2002) due to an augmented expression of endothelial and inducible nitric oxide synthase (Ye et al., 2002). An increased NO levels evoked by CSH may change the oxidative state and/or produce protein nitrosylation in type-I cells (Monteiro et al., 2005). It is possible that increased levels of NO induced by CSH may contribute to enhances the oxygen sensitivity in the CB. Iturriaga et al. (2000) reported that during steady chemosensory excitation induced by hypoxia, bolus injections of a NO donor transiently reduced chemosensory discharges. However, during normoxia the same concentration of NO increased chemosensory discharge, suggesting a dual role of NO (see also Iturriaga, 2001). Endothelin-1 (ET-1) is also present in the CB. Chronic hypoxia for 2 weeks increases the ET-1 levels and the expression of the ETA receptor in rat type-I cells (Chen Y. et al., 2002). ET peptides have a proliferative effect in the CB stimulating cellular proliferation in CB primary cultures trough the ETA receptor activation (Paciga et al., 1999). In the rabbit CB superfused *in vitro*, Chen et al. (2000) found that ET-1 did not modify basal CB chemosensory discharge, but potentiates the response induced by hypoxia. In addition, Chen Y. et al. (2002) found that ET-1 increases Ca^2+^ acting on L-type Ca^2+^ channels in rabbit type-I cells. Although, ET may enhance the CB response to hypoxia in a CB preparation devoid of vascular effects, the excitatory effect of ET-1 on CB chemoreception is most likely mediated by its vasoconstrictor effect (Rey and Iturriaga, 2004). Chen et al. (2007) found that the enhanced basal and hypoxic evoked chemosensory activity following CSH was significantly reduced by concurrent treatment with the ETA receptors blocker bosentan. Thus, it is plausible that CSH-induced CB acclimatization would be partially mediated by ETA receptors.

### Changes in Levels and Secretion of DA, ACh, ATP, or Their Receptors

Carotid body type-I cells contains high levels of dopamine (DA), thus tyrosine hydroxylase (TH) is a conventional marker for the identification of type-I cells (Chou et al., 1998). CSH enhances both, the expression and activity of TH in CBs (Hanbauer, 1977; Czyzyk-Krzeska et al., 1992); as a consequence, there is an increased DA content. It is worth to mention that even though DA is released in response to acute hypoxia, its role in CB neurotransmission remains controversial (Fidone et al., 1990; Alcayaga et al., 2006). In most species, the evidence strongly suggests an inhibitory role for DA on CB chemoreception. It has been proposed that DA is involved in an auto-regulatory mechanism mediated by D2 dopaminergic receptors expressed in type-I cells (Fidone et al., 1990). Some evidences suggesting that the inhibitory role of DA could be attenuated in CBs exposed to CSH, leading to the increased chemoreactivity (Tatsumi et al., 1995). However, this is in sharp contrast with the increased TH activity during chronic hypoxia. Zhang et al. (2017) proposed that adenosine and DA control CB chemosensory discharge acting on petrosal neurons via their opposing actions on the hyperpolarization-activated, cyclic nucleotide-gated (HCN) cation current (Ih). By using a functional *in vitro* preparation of rat type-I cells/petrosal neurons co-cultures, they found that adenosine enhanced Ih in petrosal neurons acting on A2a receptors, while DA had the opposite effect via D2 receptors. Indeed, adenosine modified the Ih activation curve and increased firing frequency, while DA caused a hyperpolarizing shift in the curve and decreased the firing frequency (Zhang et al., 2017).

Acetylcholine and ATP have been proposed as excitatory transmitters in the CB, acting on nicotinic and P2X receptors, respectively (Zhang et al., 2000; Varas et al., 2003; Meza et al., 2012). In response to CSH, an increase of nicotinic acetylcholine receptor (nAChR) expression in chemosensory afferents has been reported (He et al., 2005). Paradoxically, nAChRs blockers does not revert the CB hyperreactivity evoked by chronic hypoxia (Bisgard, 2000). Regarding to the role played by ATP, it seems that CSH has no effect on P2X receptors expression in both, type-I cells and postsynaptic terminals of petrosal neurons. However, administration of P2X receptor blockers reduced the enhanced chemosensory response to acute hypoxia induced by CSH (He et al., 2006). Therefore, reports suggest that ACh and ATP may play a role in the hyperreactivity of the CB induced by CSH. Hence, it seems that chronic hypoxia enhances both the excitatory (purinergic and cholinergic) and inhibitory (dopaminergic) chemoreception pathways, in agreement with the proposal stating that the balance between excitatory and inhibitory transmitters is more relevant during chronically hypoxic conditions rather than normoxia (Prabhakar, 2006).

### Increase in the Proportion of Na^+^ Currents and a Decrease in K^+^ Currents

In type-I cells of neonatal rats subjected to long-lasting chronic hypoxia there is an increase in the proportion of Na^+^ currents and a decrease in oxygen-sensitive K^+^ current density (Hempleman, 1995). Likewise, Cáceres et al. (2007) found that rat CB hyperreactivity induced by CSH correlated with an increased expression of voltage-gated Na^+^ channel Nav1.1 subunit. Carpenter et al. (1998) found that exposing adult rats to 10% O_2_ for 3 weeks significantly reduced K^+^ current density with no change in voltage-gated K^+^ current amplitudes. However, rabbit CBs cultured in 5% O_2_ for 2 days shows a decrease in expression of the Kv3.4 subunit of the voltage-gated K^+^ channel (Kääb et al., 2005), suggesting a species-specific effect on Kv-conductance.

Additionally, neonatal rat type-I cells exposed to hypoxia (9–14 days, 10% O_2_) shows a decreased charybdotoxin-sensitive K^+^ current, suggesting the downregulation of the maxi-K channel (Wyatt et al., 1995). It is worth to mention that in this paradigm, the rats have “blunted” rather hyperreactive responses to acute hypoxia (Wyatt et al., 1995). In culture, human maxi-K channels show an increased calcium sensitivity after 72 h of hypoxia. This is probably due to the reported threefold increase in maxi-K β-regulatory subunit expression induced by CSH (Hartness et al., 2003). However, cultured type-I cells from neonatal rat subjected to CSH, express normal levels of maxi-K and TASK-1 channels (Nurse and Fearon, 2002). Additionally, cultured CBs cells exposed to chronic hypoxia for 2 weeks showed an increase in Na^+^ currents, but no changes in Ca^2+^ currents were reported (Stea et al., 1995), findings confirmed later in a paradigm of adult rats exposed to CSH (Peers et al., 1996).

We found an enhanced inhibition of the TASK-like current in neonatal rat type-I cells in cultured exposed to hypoxia for 2 days (Ortiz et al., 2007). While acute hypoxia (1% O_2_) evokes a ∼70% inhibition of the TASK-like current in control conditions, after 48 h of hypoxia current inhibition reaches ∼90%. Importantly, these results were later reproduced (Ortiz et al., 2013) in an animal model for chronic *intermittent* hypoxia suggesting a common preserved mechanism of CB adaptation to hypoxic environments.

The evidence suggests that regulation of the expression and/or function of ionic currents are important adaptive mechanisms for the CB subjected to chronic hypoxia. Nevertheless, with the exception of the work of Cáceres et al. (2007), there is no physiological evidence that the CSH protocols used in above mentioned studies (Hempleman, 1995; Stea et al., 1995; Carpenter et al., 1998) really potentiated the CB chemosensory responses to acute hypoxia. On the contrary, most of them used long-term CSH exposition (>2 weeks) which is associated with an attenuation rather than a potentiation of CB chemosensory responses to acute hypoxia (Tatsumi et al., 1991). Hence, no convincing conclusion can be draw.

### Increased Activity of AMP-Dependent Kinase and Protein Kinase C (PKC)

The activities of maxi-K and TASK channels are modulated by cell metabolism. Wyatt et al. (2007) found that AMP-dependent kinase (AMPK) is colocalized with both maxi-K and TASK channels in rat type-I cells. They found that AMPK activation inhibits those K^+^ currents triggering membrane depolarization, whereas AMPK-antagonist application prevents the depolarization induced by hypoxia (Wyatt et al., 2007).

TASK channel current is activated by intracellular nucleotides, such as ATP (Varas et al., 2007). At physiological concentrations (1–3 mM) ATP strongly promotes channel opening. Accordingly, in absence of ATP, TASK channel activity decreases in ∼50 % in inside-out patches (Williams and Buckler, 2004; Varas et al., 2007). Thus, ATP production seems to be central in regulating the activity of TASK-like and other potassium channels such as maxi-K (Williams and Buckler, 2004; Varas et al., 2007; Holmes et al., 2018). Even though some reports postulate a null or small effect of ATP on these conductances (Xu et al., 2005) one of the earliest proposition, the “metabolic hypothesis”, states that CB oxygen sensing is mediated by oxidative phosphorylation, supported by the fact that virtually all metabolic poisons or oxidative phosphorylation inhibitors induce CB type-I cell excitation (Mulligan and Lahiri, 1982; Buerk et al., 1994; Wilson et al., 1994; Buckler and Turner, 2013). This response is normally triggered by potassium conductance(s) inhibition leading to the depolarization of the type-I cell membrane, reinforcing the notion that low PO_2_ trigger potassium current blockage (**Figure 1**, see also Holmes et al., 2018). In this regard, it has been postulated that CB-mitochondria express a particular subtype of *cytochrome a3* – the functional core of mitochondrion complex IV – which is at least seven times more sensitive to a drop in the PO_2_, further supporting a pivotal role of mitochondrial metabolism on CB-type-I cell excitation (for a full revision on this matter see Holmes et al., 2018 in this same research topic).

Therefore, in addition to changes in the expression and intrinsic function of ion channels, the fact that cellular metabolism is modified by CSH provides a potential link between CSH and the type-I cell membrane excitability. It is known that CSH increases the cytosolic AMP/ATP ratio in type-I cells. Thus, several enzymatic systems can be activated, including AMPK (Laderoute et al., 2006). Likewise, CSH induces an increase in cAMP levels in CB cells (Nurse et al., 1994). Protein kinase C (PKC) activity is enhanced by chronic hypoxia in several tissues (Zhang et al., 2004; Neckar et al., 2005), whereas the protein kinase A (PKA) activity decreases (Kobayashi et al., 1998). Increased PKC activity in response to acute hypoxia (Summers et al., 2000) was associated to the regulation of L-type Ca^2+^, maxi-K channels and background K^+^ channels in type-I cells (Peers and Carpenter, 1998; Summers et al., 2000; Zhang et al., 2003). Since PKC inhibits TASK channel activity, whereas PKA enhanced it (Zilberberg et al., 2000; Besana et al., 2004) these pathways are well-suited as potential effectors of CSH induced-changes.

### Activation of Hypoxia-Inducible Transcription Factors (HIFs)

The cellular long-term response to hypoxia is driven by changes in the expression of several genes to cope with the new hypoxic environment. These cellular transcriptional responses are largely dependent on the activation of the so-called hypoxia-inducible transcription factors (Shimoda and Semenza, 2011). HIFs are composed by a HIFα subunit (mainly 1α and 2α), regulated by hypoxia and a constitutive β subunit (HIF-1β). Under normoxic conditions, HIFα is continuously degraded through hydroxylation modification catalyzed by a set of oxygen sensitive prolyl-hydroxylases enzymes. During hypoxia, the activity of prolyl-hydroxylases are dramatically reduced, allowing the translocation of HIFα to the cell nuclei where it forms an heterodimer with HIF-1β. Following translocation and dimerization, the HIF-1α/HIF-1β complex bind to hypoxia response elements (HRE) present in the DNA to regulate gene expression (Shimoda and Semenza, 2011). In the CB, the contribution of HIF-1α and HIF-2α has been largely studied in the setting of intermittent hypoxia; much less it is known in the context of CSH. Nevertheless, it has been shown that activation of the HIF-1α pathway promotes the up-regulation of pro-oxidant enzymes (i.e., NADPH) in the CB. On the contrary, intermittent hypoxia reduced the expression of HIF-2α within the CB, resulting in a significant down-regulation of antioxidant enzymes (i.e., SOD) levels being the outcome an increase ROS formation within CB glomus cells, increasing both intracellular Ca^2+^ and neurotransmitter release (Prabhakar and Semenza, 2016). The specific contribution of HIF-1α and HIF-2α pathways on the sensitization of the CB to hypoxia under CSH remains to be fully determined. Nevertheless, it is possible to speculate that activation of HIF-1α and inhibition of HIF-2α pathways may occur in the CBs of animals exposed to sustained hypoxia.

We have here discussed some of the mechanisms explaining CB chemosensory hyperreactivity under CSH. This exacerbated activity has systemic consequences inducing the adaptation to CSH. In this regard, a central aspect is the CB-mediated autonomic function and even though the neural mechanism associated to autonomic responses to CSH are not completely elucidated, it has been described that CB type-I cells could be involved. During acute hypoxic exposure, excitatory inputs from the CB activate neural pathways in the brainstem increasing minute ventilation and sympathetic outflow (Barros et al., 2002; Braga et al., 2007). However, during CSH the baseline minute ventilation increases in a time-dependent manner (ventilatory acclimatization to hypoxia), probably related to the changes in oxygen sensitivity of the CB as well as the neural network in the brainstem related to sympathetic response to hypoxia (Powell et al., 2000; Powell, 2007). It has been proposed that hypoxia-mediated sympathetic activation act as a compensatory mechanism to increase oxygen supply to critical organs through regulating cardiac output and vascular conductance (Leuenberger et al., 1991; Calbet, 2003). Indeed, exposure of healthy humans to CSH produces a significant increase in circulating norepinephrine levels reflecting increases in sympathetic outflow during hypoxic exposure (Calbet, 2003; Hansen and Sander, 2003). In addition, it has been shown that rats subjected to 24 h of FiO_2_ 10% displayed an increase of pre-sympathetic neurons activation located in the rostral aspect of the ventral medullary surface (RVLM). Furthermore, these set of neurons showed a further increase activation during the late expiratory phase during hypoxia stimulation suggesting a potential role on regulating the neural circuitry related to the respiratory-sympathetic coupling observed during CSH (Moraes et al., 2014). Interestingly, previous studies from our group showed that chronic activation of the CB afferent input to the brainstem using episodic hypoxic stimulation induces the activation of RVLM neurons (Del Rio et al., 2016). Therefore, it is plausible to hypothesize that during CSH, activation of the CB may contribute to the regulation of sympathetic drive. This hypothesis remains to be determined.

## Conclusion

Potentiation of CB chemosensory responses is an absolute requirement for ventilatory acclimatization. How the increased response to acute hypoxia in chronically hypoxic CBs is achieved? There are probably multiple mechanisms underlying such phenomena. In the present review we focused on evidences suggesting that type-I cells excitability and metabolism undergoes substantial changes when exposed to CSH. Interestingly, ion channels, including some involved in the generation of the chemosensory response to acute hypoxia (i.e., maxi-K and TASK channels) are tightly regulated by PKC, PKA, AMPK, and ATP. Therefore, it is possible that throughout changes in cell metabolism, in addition to modifications on ion channels expression, CSH generates changes in type-I cells excitability, ultimate leading to increased depolarization during acute hypoxia. Importantly, increased inhibition of TASK-like currents under chronic – sustained and intermittent – hypoxia provide a novel potential mechanism to explain the CB hyperreactivity (Ortiz et al., 2007, 2013). It seems clear that there is no a single mechanism as full explanation for CB hyperreactivity during CSH. Combined studies covering from cellular/molecular studies to functional approaches will help to understand the physiology of ventilatory acclimatization during chronic sustained hypoxia.

## Author Contributions

All authors contributed to writing the article. RV, RI, RDR, and FO designed and edited the paper.

## Conflict of Interest Statement

The authors declare that the research was conducted in the absence of any commercial or financial relationships that could be construed as a potential conflict of interest.
